# Early lowering of blood pressure after acute intracerebral haemorrhage: a systematic review and meta-analysis of individual patient data

**DOI:** 10.1136/jnnp-2021-327195

**Published:** 2021-11-03

**Authors:** Tom J Moullaali, Xia Wang, Else Charlotte Sandset, Lisa J Woodhouse, Zhe Kang Law, Hisatomi Arima, Kenneth S Butcher, John Chalmers, Candice Delcourt, Leon Edwards, Salil Gupta, Wen Jiang, Sebastian Koch, John Potter, Adnan I Qureshi, Thompson G Robinson, Rustam Al-Shahi Salman, Jeffrey L Saver, Nikola Sprigg, Joanna M Wardlaw, Craig S Anderson, Philip M Bath, the Blood Pressure in Acute Stroke (BASC) Investigators

**Affiliations:** 1 The George Institute for Global Health, Faculty of Medicine, University of New South Wales, Sydney, New South Wales, Australia; 2 Centre for Clinical Brain Sciences, University of Edinburgh, Edinburgh, UK; 3 Department of Neurology, Oslo University Hospital, Oslo, Norway; 4 Research and Development Department, The Norwegian Air Ambulance Foundation, Oslo, Norway; 5 Stroke Trials Unit, University of Nottingham, Queen’s Medical Centre, Nottingham, UK; 6 Stroke, Nottingham University Hospitals NHS Trust, Nottingham, UK; 7 National University of Malaysia, Kuala Lumpur, Malaysia; 8 Department of Preventive Medicine and Public Health, Fukuoka University, Fukuoka, Japan; 9 Prince of Wales Clinical School, University of New South Wales, Randwick, New South Wales, Australia; 10 Neurology Department, Royal Prince Alfred Hospital, Sydney, New South Wales, Australia; 11 Central Clinical School, the University of Sydney, Sydney, New South Wales, Australia; 12 Department of Neurology, Army Hospital Research and Referral, New Delhi, India; 13 Department of Neurology, Xijing Hospital, Fourth Military Medical University, Xi’an, China; 14 The Shaanxi Cerebrovascular Disease Clinical Research Center, Xi’an, China; 15 Department of Neurology, University of Miami Miller School of Medicine, Miami, Florida, USA; 16 Stroke Research Group, Norfolk and Norwich University Hospital, UK; 17 Norwich Medical School, University of East Anglia, UK; 18 Zeenat Qureshi Stroke Institute and Department of Neurology, University of Missouri, Columbia, MO; 19 University of Leicester, Department of Cardiovascular Sciences and NIHR Leicester Biomedical Research Centre, Leicester, UK; 20 Department of Neurology and Comprehensive Stroke Center, UCLA, Los Angeles, California, USA; 21 The George Institute China at Peking University Health Science Center, Beijing, PR China

**Keywords:** stroke, meta-analysis

## Abstract

**Objective:**

To summarise evidence of the effects of blood pressure (BP)-lowering interventions after acute spontaneous intracerebral haemorrhage (ICH).

**Methods:**

A prespecified systematic review of the Cochrane Central Register of Controlled Trials, EMBASE and MEDLINE databases from inception to 23 June 2020 to identify randomised controlled trials that compared active BP-lowering agents versus placebo or intensive versus guideline BP-lowering targets for adults <7 days after ICH onset. The primary outcome was function (distribution of scores on the modified Rankin scale) 90 days after randomisation. Radiological outcomes were absolute (>6 mL) and proportional (>33%) haematoma growth at 24 hours. Meta-analysis used a one-stage approach, adjusted using generalised linear mixed models with prespecified covariables and trial as a random effect.

**Results:**

Of 7094 studies identified, 50 trials involving 11 494 patients were eligible and 16 (32.0%) shared patient-level data from 6221 (54.1%) patients (mean age 64.2 [SD 12.9], 2266 [36.4%] females) with a median time from symptom onset to randomisation of 3.8 hours (IQR 2.6–5.3). Active/intensive BP-lowering interventions had no effect on the primary outcome compared with placebo/guideline treatment (adjusted OR for unfavourable shift in modified Rankin scale scores: 0.97, 95% CI 0.88 to 1.06; p=0.50), but there was significant heterogeneity by strategy (p_interaction_=0.031) and agent (p_interaction_<0.0001). Active/intensive BP-lowering interventions clearly reduced absolute (>6 ml, adjusted OR 0.75, 95%CI 0.60 to 0.92; p=0.0077) and relative (≥33%, adjusted OR 0.82, 95%CI 0.68 to 0.99; p=0.034) haematoma growth.

**Interpretation:**

Overall, a broad range of interventions to lower BP within 7 days of ICH onset had no overall benefit on functional recovery, despite reducing bleeding. The treatment effect appeared to vary according to strategy and agent.

**PROSPERO registration number:**

CRD42019141136.

## Introduction

Elevated blood pressure (BP) after acute spontaneous intracerebral haemorrhage (ICH) is associated with adverse clinical outcomes.^1–3^ Pooled individual participant data (IPD) analyses of two medium-to-large randomised controlled trials (RCTs) of intensive BP lowering initiated early in ICH of mild-to-moderate severity (n=3829) suggest that achieving and sustaining systolic BP levels as low as 120–130 mm Hg in the first 24 hours is safe, reduces haematoma expansion and improves functional outcome.^4^

Current guidelines advocate early intensive BP lowering to a systolic target <140 mm Hg after acute ICH, based primarily on results of the first large RCT to test this strategy, the main phase Intensive Blood Pressure Reduction in Acute Cerebral Haemorrhage Trial (INTERACT2).^5^ However, a subsequent large trial, the second Antihypertensive Treatment of Acute Cerebral Haemorrhage (ATACH-II),^6^ reported neutral findings, while smaller trials of different approaches to BP lowering in mixed stroke populations in prehospital^7–9^ and in-hospital settings^10–12^ have reported mixed results. Thus, there is ongoing uncertainty over the effects of different BP-lowering interventions on clinical outcomes, and in relation to the most plausible mechanism of effect, haematoma growth.^13–15^ Additionally, the interaction of treatment strategy, timing and agent on these effects are unknown.^16^

IPD meta-analysis is considered the gold standard for synthesising evidence from RCTs.^17^ The aim of the international Blood pressure in Acute Stroke Collaboration (BASC) is to perform detailed analyses of pooled IPD from RCTs of BP management after acute stroke.^18 19^ Herein, we present results pertaining to the effects of BP-lowering interventions on outcomes after acute ICH, with a focus on determining whether there is any modification of the effect by patient characteristics, or by the strategy, timing or agent of the BP-lowering intervention.

## Methods

### Search strategy and selection criteria

We performed a systematic review according to a prespecified protocol to identify RCTs that assessed the effects of different BP-lowering strategies during the acute phase (within 7 days) of stroke.^20^ We identified eligible studies in the Cochrane Central Register of Controlled Trials, EMBASE and MEDLINE databases from inception to 23 June 2020, and in the reference lists of published systematic and ad hoc reviews using a comprehensive search strategy, limited to humans, combining terms for ICH, BP-lowering interventions and RCTs, with no language restrictions.^20^

We included trials that involved adults (>18 years) with acute primary spontaneous ICH (<7 days from onset); randomised participants to fixed active agent or intensive, titrated target-based BP-lowering interventions with oral, sublingual, transdermal or intravenous agents, in single or combination therapy versus placebo or contemporaneous guideline BP management; and recorded clinical and/or radiological outcomes.

Two authors screened titles and abstracts, and assessed full-text articles for eligibility against the inclusion criteria. We sent our protocol and a letter of invitation to investigators of eligible studies, inviting them to join the BASC and share IPD. We followed with an invitation to join (online or in-person) BASC collaborator meetings. To ensure transparency, collaborators sharing data with BASC were asked to sign a data transfer agreement for the predefined and appropriate use of their data according to our protocol.

### Data management

We checked IPD with published results to ensure data were complete and transferred without error; queries were resolved with individual trial investigators. We harmonised trial datasets according to agreed nomenclature. Two minimum datasets for primary analyses were developed in Sydney and Nottingham; covariable adjustment included trial (treatment allocation, time from symptom onset to randomisation), demographic (age and sex) and baseline clinical (stroke severity assessed by National Institutes of Health Stroke Scale [NIHSS] score^21^) characteristics. Radiological characteristics were desirable for analyses of haematoma growth (time from symptom onset to diagnostic brain scan, haematoma volume on baseline and repeat imaging 24 hours after onset assessed by ABC/2^22^ or semiautomated volumetric methods.^23^

### Outcomes

The primary outcome was functional status defined by the ordinal distribution of modified Rankin scale (mRS) scores (which range from 0 [no symptoms] to 6 [death]) at the end of trial follow-up (usually 90 days). Secondary outcomes were: (1) death or dependency (3–6 on the mRS); (2) death or severe dependency (4–6 on the mRS) and (3) all-cause death. Radiological outcomes were: (1) absolute (≥6 mL) and (2) proportional (≥33%) haematoma growth at 24 hours.^24^ Safety outcomes were: (1) early neurological deterioration (as defined by each individual trial); (2) symptomatic hypotension (as defined by each individual trial) and (3) other any serious adverse event (SAE), as defined by individual trial, to include those fatal, non-fatal and treatment related.

### Data analysis

We performed primary analysis using the intention-to-treat dataset from each trial, with a one-stage approach to IPD meta-analysis.^25^ The one-stage approach provides additional statistical power and flexibility by combining all IPD into a single meta-analysis, and permits subgroup analyses according to individual characteristics of interest (further information in online supplemental table 1). We excluded patients without the minimum dataset for covariable adjustment. Descriptive statistics are described as mean (SD) or median (IQR) for continuous data, or frequency (percentage) for categorical data, and Kruskal-Wallis or χ^2^ tests are used to make comparisons.

10.1136/jnnp-2021-327195.supp1 Supplementary data

We used generalised linear mixed models with prespecified covariables (age, sex, NIHSS score, time from symptom onset to randomisation), and the source trial as a random effect to account for clustering. We chose to adjust for the NIHSS score as it was previously shown to be a better discriminator of poor outcome after acute ICH.^26^ Analyses of ordinal and binary outcome variables are presented as OR with 95% CIs. We checked the proportional odds assumption using the likelihood ratio test before undertaking ordinal analyses of outcomes on the mRS. Analyses of continuous outcome variables are presented as mean differences with 95% CIs.

In order to test the modifying effects of patient characteristics, and the strategy, timing and agent used in the BP-lowering interventions on outcomes, we performed the following subgroup analysis with an interaction term in models to test heterogeneity: age (≤65 vs >65 y), sex, stroke severity (NIHSS score ≤10 vs >10), baseline haematoma volume (≤10 vs >10 mL), BP-lowering strategy (fixed active agent vs titrated to intensive target), time from symptom onset to randomisation (<2 vs 2–6 vs 6–24 vs ≥24 hour), trial setting (prehospital vs in-hospital), and most frequently used BP-lowering agent in the treatment group (renin-angiotensin system blocker vs α-adrenoreceptor blocker and β-adrenoreceptor blocker vs calcium channel blocker vs nitrate vs magnesium). We grouped α-adrenoreceptor blockers and β-adrenoreceptor blockers together as included agents (intravenous urapidil and labetalol) had varying degrees of α-adrenoreceptor and β-adrenoreceptor blocking activity, although their effect on BP is primarily achieved through α-adrenoreceptor blockade.^27 28^ For subgroup analyses with three or more prespecified subgroups, we explored any patterns that emerged in dichotomised subgroups to increase statistical power.

We assessed all eligible studies for bias using the Cochrane Collaboration tool (https://methods.cochrane.org/bias/) and used the robvis risk of bias tool to generate visual summaries.^29^ We assessed publication bias by: (1) a visual inspection for funnel plot asymmetry and (2) with Egger’s regression test.

We did unadjusted one-stage and adjusted two-stage sensitivity analyses to test the robustness of our findings. Due to differences in study designs and outcome reporting for studies that did not share IPD (of 34 studies that did not share IPD, 2 studies reported mRS outcome data; 28 studies did not and in 2 studies it was unclear, online supplemental table 3), we were unable to do the following prespecified analysis for all studies: two-stage analysis of the primary outcome using study-level data and assessment of publication bias.

To assess confounding from anticoagulant use (64 of 11716 [3.7%] vs 43 of 1731 [2.5%] with available data in active/intensive versus placebo/guideline groups, respectively),^25^ we did a post hoc sensitivity analysis of the effect of BP lowering on absolute and relative haematoma growth at 24 hours with additional adjustment for anticoagulant use. We observed a pattern emerging for a potential interaction effect from the timing of the BP-lowering intervention across four prespecified subgroups, so we did a post hoc assessment using a dichotomous grouping (<6 vs ≥6 hours) to maximise statistical power. We also assessed the three-way interaction between treatment allocation and timing of the BP-lowering intervention by (1) strategy and (2) agent. In view of our finding that treatment with intravenous magnesium had no effect on BP control during 1–24 hours compared with placebo, we did post hoc analysis excluding the trial of intravenous magnesium versus placebo.

## Results

**Figure 1:**
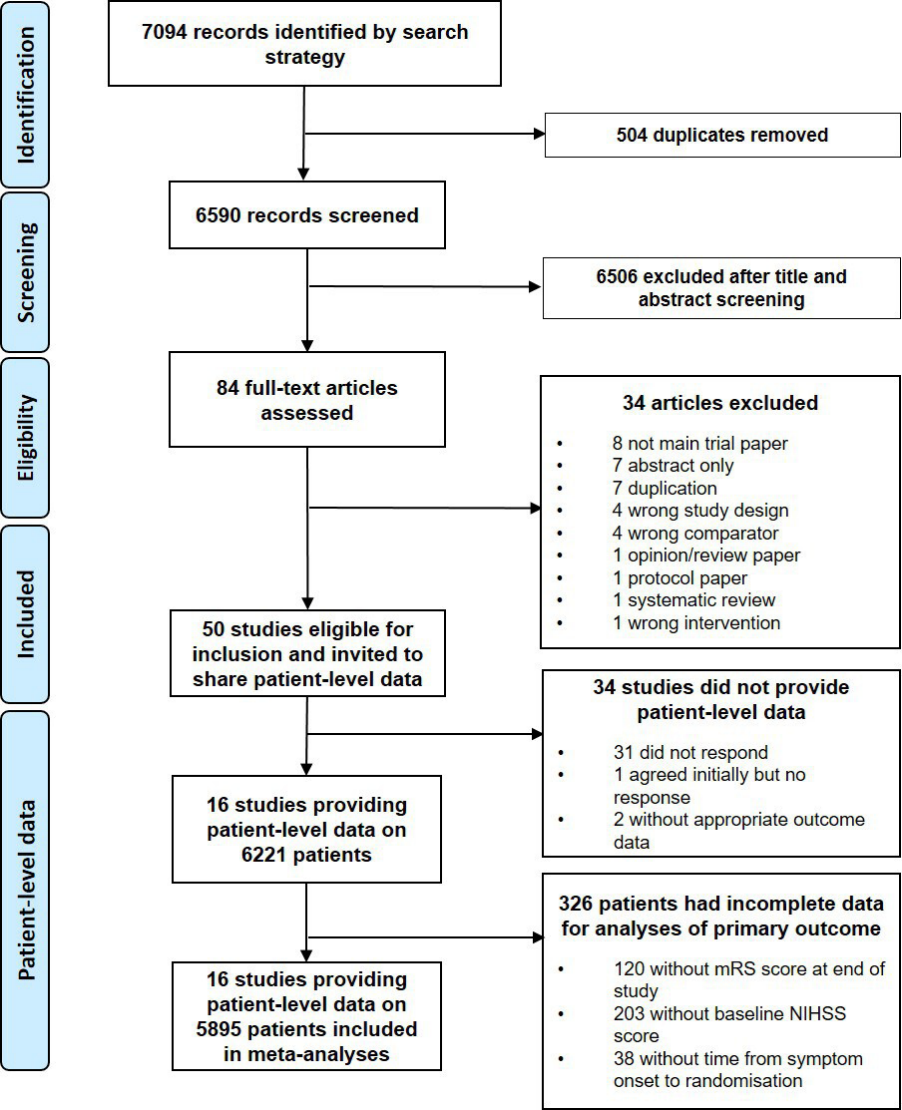
Study selection. mRS, modified Rankin scale; NIHSS, National Institutes of Health Stroke Scale.

**Figure 2:**
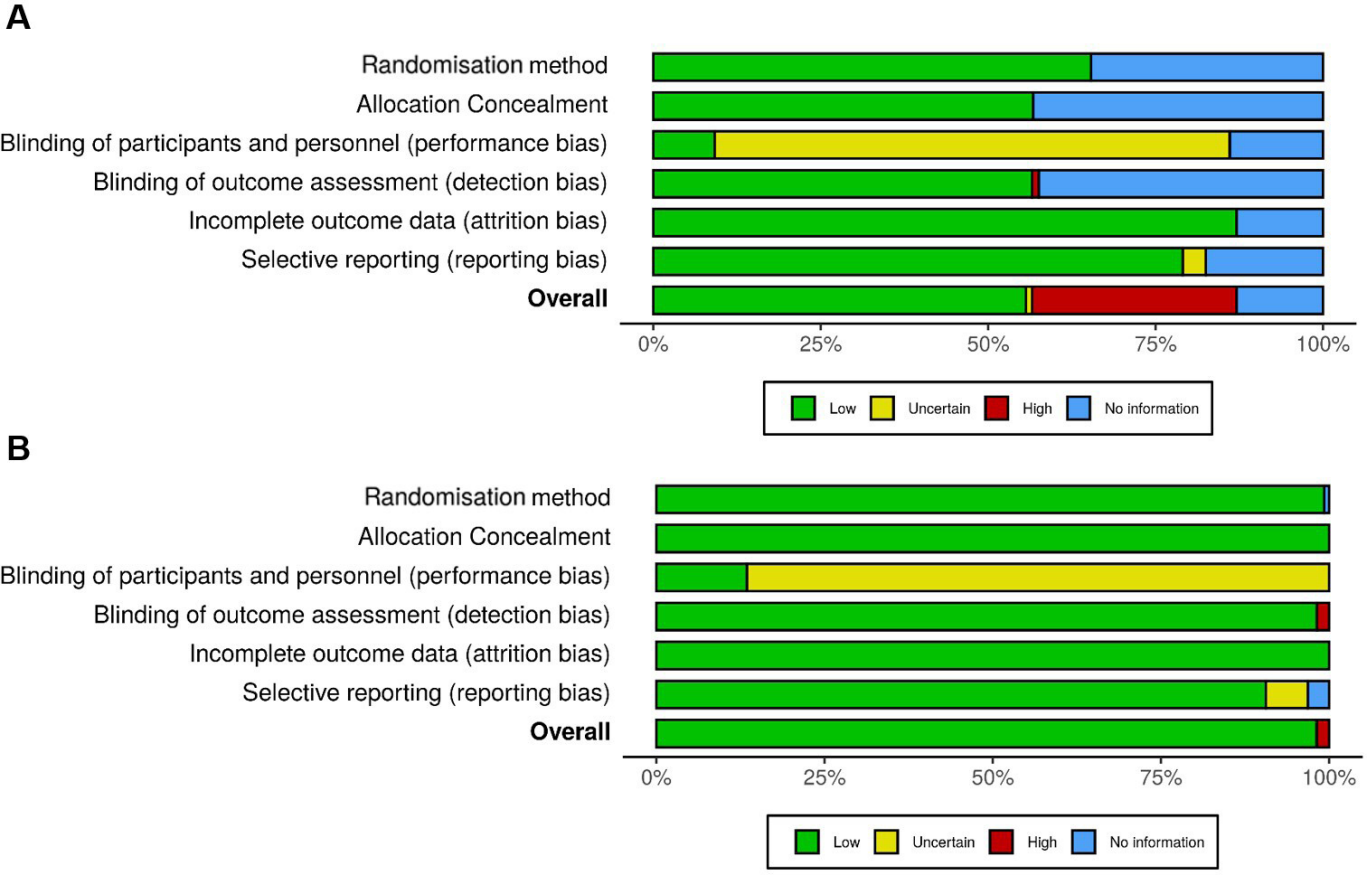
Weighted bias plot: (A) all studies and (B) studies that shared patient-level data. Complete bias assessment for individual studies and the design characteristics of studies that did and did not share patient-level data are summarised in the online supplemental materials.

We screened 7094 studies identified through our search strategy, assessed 84 full texts for eligibility, invited authors of 50 eligible studies to share data and obtained IPD from 16 (32.0%) studies (figure 1). This amounted to 6221 of 11 494 (54.1%) participants recruited to eligible studies; the characteristics of studies that did and did not share IPD are summarised in online supplemental tables 2 and 3. There were no issues with the integrity of IPD, and the studies that shared IPD were at low risk of bias (figure 2, online supplemental table 4). We found no evidence of publication bias among included studies on a visual inspection of the funnel plot and using Egger’s regression test (p=0.11).

Table 1 summarises the baseline characteristics of included participants with acute ICH, of whom 3494 (56.2%) were recruited in Asia, 1696 (27.2%) in Europe/Australia and 1031 (16.6%) in the Americas. Baseline demographic and clinical characteristics were well balanced between 3121 participants randomly assigned to active/intensive BP management, and 3100 to placebo/guideline BP management. Overall, their mean age was 64.2 (SD 12.9) years and 2266 (36.4%) were female, with a median level of baseline neurological impairment, defined by NIHSS score of 11 (range 0–42, IQR 7–16). Overall mean systolic and diastolic BP at randomisation were 177.3 mm Hg (SD 20.3) and 100.0 mm Hg (SD 15.7), respectively, and the median time from onset to randomisation to various BP-lowering strategies was 3.8 hours (IQR 2.6–5.3). The median haematoma volume on the diagnostic CT brain scan was 10.7 mL (IQR 5.2–20.7).

**Table 1:** Characteristics of included patients by randomly allocated treatment group

Characteristics	Randomly allocated treatment group		Standardised difference*
	Active/intensive (N=3121)	Placebo/guideline (N=3100)	
Age, years	64.0 (13.0)	64.3 (12.9)	0.028
Sex (female)	1146/3121 (36.7)	1120/3100 (36.1)	0.012
Geographical region†			
Americas	520/3121 (16.7)	511/3100 (16.5)	0.026
Asia	1734/3121 (55.6)	1760/3100 (56.8)	
Europe/Australia	867/3121 (27.8)	829/3100 (26.7)	
SBP at randomisation, mm Hg	177.3 (20.1)	177.4 (20.5)	0.007
DBP at randomisation, mm Hg	99.9 (15.5)	100.1 (15.8)	0.014
NIHSS score	11.0 (7.0 to 16.2)	11.1 (7.0 to 16.0)	0.028
GCS score	14.0 (12.0 to 15.0)	14.0 (13.0 to 15.0)	0.005
History of hypertension	2219/3023 (73.4)	2179/2998 (72.7)	0.016
History of diabetes mellitus	405/2987 (13.6)	364/2975 (12.2)	0.039
History of stroke	513/3014 (17.0)	503/2994 (16.8)	0.006
History of ischaemic heart disease	273/2815 (9.7)	275/2827 (9.7)	0.001
Current use of antihypertensive drugs	1194/2653 (45.0)	1183/2652 (44.6)	0.008
Current antiplatelet therapy	163/1734 (9.4)	178/1741 (10.2)	0.028
Current anticoagulation	64/1716 (3.7)	43/1731 (2.5)	0.072
Haematoma volume, mL	10.6 (5.1 to 20.5)	10.7 (5.4 to 20.7)	0.037
Haematoma location			
Lobar	249/1942 (12.8)	257/1921 (13.4)	0.017
Basal ganglia/deep	1589/1942 (81.8)	1547/1921 (80.5)	0.033
Infratentorial/posterior fossa	104/1942 (5.4)	117/1921 (6.1)	0.032
Intraventricular haemorrhage	815/2729 (29.9)	808/2691 (30.0)	0.004
Time from symptom onset to randomisation, hour	3.8 (2.5 to 5.4)	3.8 (2.6 to 5.2)	0.018
Process of care variables			
DNAR	133/2177 (6.1)	109/2200 (5.0)	0.051
Intubation	204/2324 (8.8)	181/2338 (7.7)	0.038
Neurosurgery	133/2322 (5.7)	142/2338 (6.1)	0.015

Data are numbers (%), mean (SD) or median (IQR).

*A standardised difference of 10% (or 0.1) is equivalent to a p=0.05.

†Geographical region denotes the country in which patients were treated.

DBP, diastolic blood pressure; DNAR, do not attempt resuscitation order; GCS, Glasgow Coma Scale; NIHSS, National Institutes of Health Stroke Scale; SBP, systolic blood pressure.

There were 5895 (94.8%) patients with complete IPD for adjusted analysis of the primary outcome. There were 326 (5.2%) patients without the minimum required data excluded from analyses: 120 (2.0%), 203 (3.3%) and 38 (0.6%) were missing data on mRS scores, NIHSS scores, and time from symptom onset to randomisation, respectively. Due to differences in the aims and designs of trials sharing IPD, there were 2510 (43.1%) patients with IPD and a CT scan reassessment at 24 hours for analysis of the key radiological outcome of haematoma growth.

Compared with patients who received placebo/guideline BP management, BP was significantly lower in patients who received active/intensive management: mean differences in mean systolic BP were −7.5 mm Hg (95% CI −8.6 to −6.3; p<0.0001), –12.1 mm Hg (95% CI −13.0 to −11.2; p<0.0001) and −7.3 mm Hg (95% CI −8.2 to −6.5; p<0.0001) within 1 hour, 1–24 hours and during 2–7 days, respectively; mean differences in diastolic BP in the same time epochs were −3.8 mm Hg (95% CI −4.8 to −2.8; p<0.001), –5.3 mm Hg (95% CI −6.0 to −4.5; p<0.001) and −3.9 mm Hg (95% CI −4.6 to −3.3; p<0.001). Postrandomisation BP control during 1–24 hours varied across individual studies in a two-stage approach: compared with their respective controls, larger mean reductions in systolic BP were achieved in patients randomised to intensive, titrated target-based BP-lowering strategies compared with patients randomised to fixed active agent-based BP-lowering strategies.

**Figure 3:**
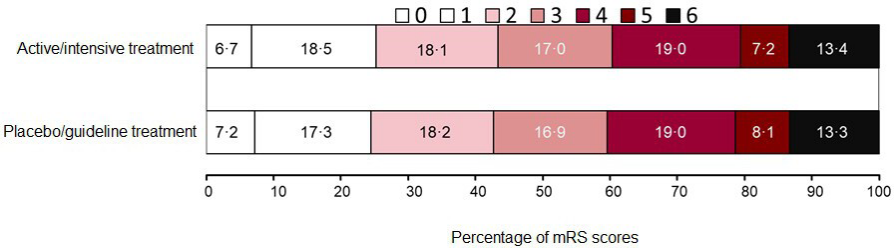
Distribution of scores on the modified Rankin Scale (mRS) in active/intensive versus placebo/guideline groups after acute intracerebral haemorrhage.

Table 2 shows the randomised treatment effect of BP-lowering interventions on the primary, secondary, safety and radiological outcomes, using a one-stage approach. The proportional odds assumption was fulfilled using the likelihood ratio test for ordinal analyses of the primary outcome of functional status assessed across the 7-levels of the mRS. Overall, there was no effect of active/intensive BP management on the distribution of mRS scores at the end of trial follow-up (mRS data for 5830 patients from 12 studies at 90 days, 271 patients from 1 study at 180 days): adjusted OR for unfavourable shift in mRS scores 0.97 (95% CI 0.88 to 1.06; p=0.50), table 2 and figure 3. This finding was consistent across various standard cut points on the mRS that reflect greater disability (table 2), in prespecified sensitivity analyses using adjusted two-stage and unadjusted one-stage models, and after adjustment for Glasgow Coma Scale score rather than NIHSS score (online supplemental table 5). There was no effect of treatment allocation on death: adjusted OR 1.01 (95% CI 0.85 to 1.20; p=0.91).

**Table 2:** Effect of active/intensive versus placebo/guideline blood pressure lowering interventions on outcomes after acute intracerebral haemorrhage (ICH)

Outcome	Randomly allocated treatment group		Adjusted OR* (95% CI)	P value
	Active/intensive	Placebo/guideline		
Primary: unfavourable shift in modified Rankin Scale (mRS) scores	/3062	/3039	0.97 (0.88 to 1.06)	0.50
0	206 (6.7)	219 (7.2)		
1	566 (18.5)	525 (17.3)		
2	555 (18.1)	553 (18.2)		
3	521 (17.0)	514 (16.9)		
4	582 (19.0)	578 (19.0)		
5	221 (7.2)	245 (8.1)		
6	411 (13.4)	405 (13.3)		
Secondary	/3062	/3039		
Dependency or death (mRS 3–6)	1735 (56.7)	1742 (57.3)	0.95 (0.84 to 1.08)	0.42
Severe dependency or death (mRS 4–6)	1214 (39.7)	1228 (40.4)	0.95 (0.84 to 1.08)	0.41
Death	411/3111 (13.2)	405/3087 (13.1)	1.01 (0.85 to 1.20)	0.91
Safety outcomes				
Any SAE	781/3000 (26.0)	727/2982 (24.4)	1.13 (0.99 to 1.29)	0.07
Neurological deterioration†	304/2911 (10.4)	300/2913 (10.3)	1.02 (0.86 to 1.22)	0.81
Severe hypotension†	25/2831 (0.9)	15/2840 (0.5)	1.73 (0.89 to 3.37)	0.11
Cardiac SAE	69/2978 (2.3)	70/2980 (2.3)	1.01 (0.72 to 1.42)	0.96
Renal SAE	34/2978 (1.1)	28/2980 (0.9)	1.26 (0.75 to 2.09)	0.38
Haematoma growth at 24 hours**‡**				
Mean growth (95% CI), mL	3.2 (2.5 to 3.9)	4.3 (3.4 to 5.2)	Absolute difference: −1.10 (−2.22 to 0.01)	0.05
Absolute growth ≥6 mL	212/1280 (16.6)	252/1230 (20.5)	0.75 (0.60 to 0.92)§	0.007
Relative growth ≥33%	296/1280 (23.1)	326/1230 (26.5)	0.82 (0.68 to 0.99)§	0.03

*Model adjusted for age, sex, National Institutes for Health Stroke Scale score, time from onset to randomisation and trial (random effect); all patients followed up for 90 days except those in the Scandinavian Candesartan Acute Stroke Trial who were followed for 6 months.

†Treatment related, as defined by each trial.

‡Trials contributing data for analyses of haematoma growth at 24 hours: INTERACT1&2, ICH-ADAPT, ATACH-II, FAST-MAG.

§Model adjusted for age, sex, baseline haematoma volume and time from symptom onset to randomisation.

CI, confidence interval; mRS, modified Rankin Scale; OR, odds ratio; SAE, serious adverse event.

**Figure 4:**
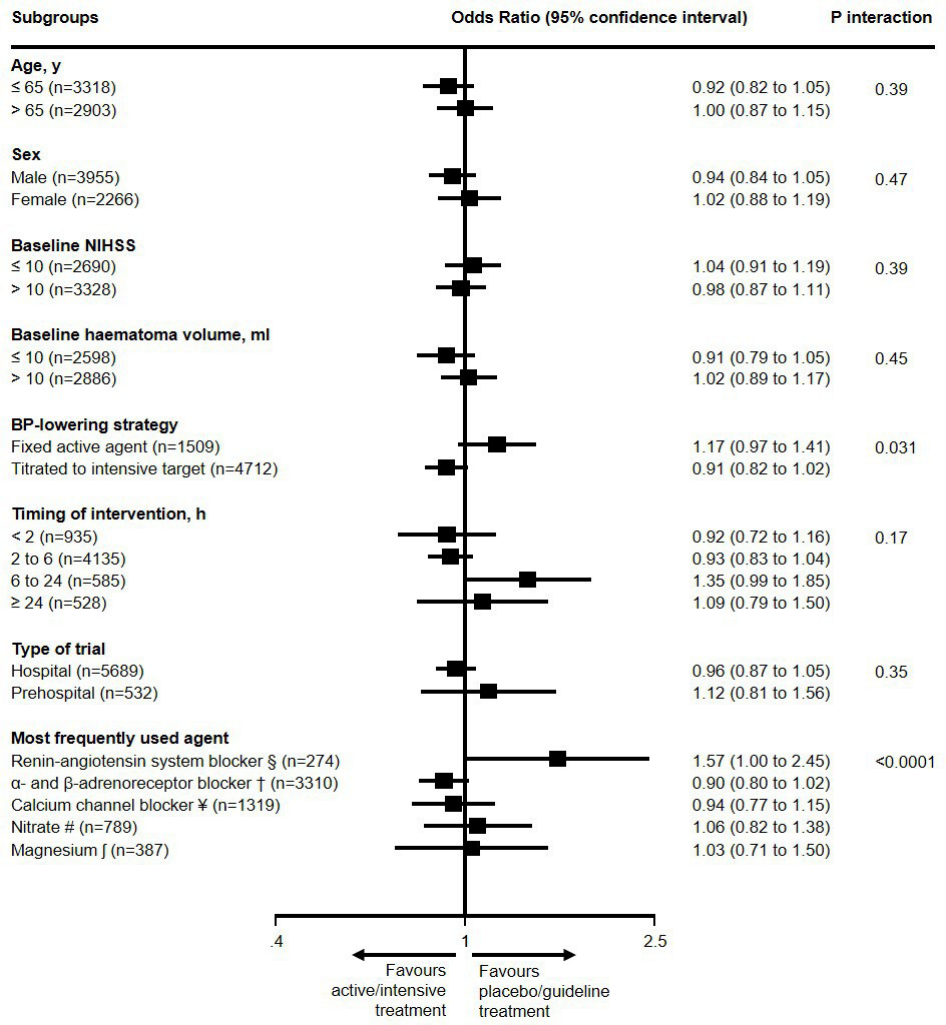
Effects of blood pressure (BP) lowering interventions in active/intensive versus placebo/guideline groups after acute intracerebral haemorrhage on the primary outcome in subgroups. Model adjusted for age, sex, National Institutes for Health Stroke Scale (NIHSS) score and time from symptom onset to randomisation. §SCAST; †INTERACT1&2, ICH-ADAPT; ¥ ATACH-II, CHASE, Koch 2018, VENUS; # GTN1&2, ENOS, RIGHT1&2; ʃ FAST-MAG.

We assessed heterogeneity in the treatment effect on the primary outcome across prespecified subgroups (figure 4). A significant interaction existed for BP-lowering strategy (p_interaction_=0.031): there was a non-significant increase in the odds for unfavourable shift in mRS scores in patients treated with fixed active BP-lowering agents (adjusted OR 1.17, 95% CI 0.97 to 1.41), compared with a non-significant decrease in the odds for unfavourable shift in mRS scores for patients treated with BP lowering titrated to an intensive target (0.91, 95% CI 0.82 to 1.02). Additionally, heterogeneity was noted between different BP-lowering agents (p_interaction_ <0.0001): patients treated with renin–angiotensin system blockers had significantly increased odds for unfavourable shift in mRS scores (adjusted OR 1.57, 95% CI 1.00 to 2.45), while patients treated with α-adrenoreceptor and β-adrenoreceptor blockers and calcium channel blockers had non-significant reductions in the odds for unfavourable shift in mRS scores (adjusted OR 0.90, 95% CI 0.80 to 1.02, and adjusted OR 0.94, 95% CI 0.77 to 1.15, respectively). There was no significant interaction according to timing of BP-lowering intervention. The results did not change in a post hoc sensitivity analysis where patients from a trial of intravenous magnesium versus placebo were excluded.

In post hoc analysis, there was no evidence of heterogeneity in the effect of BP-lowering interventions initiated earlier (<6 hours) compared with later (≥6 hours) after the onset of symptoms (OR for unfavourable shift in mRS scores: 0.93, 95% CI 0.84 to 1.03, vs OR 1.20, 95% CI 0.96 to 1.49; p_interaction_=0.081). Three-way interactions between treatment allocation, the timing of the BP-lowering intervention and (1) the strategy (fixed active agent vs intensive target) and (2) the agent were not statistically significant (p_interaction_=0.652 and p_interaction_=0.571, respectively).

Table 2 also shows there was no difference in the number of patients with any SAE between active/intensive and placebo/guideline groups, nor in neurological deterioration, severe hypotension or cardiac or renal SAE.

**Figure 5:**
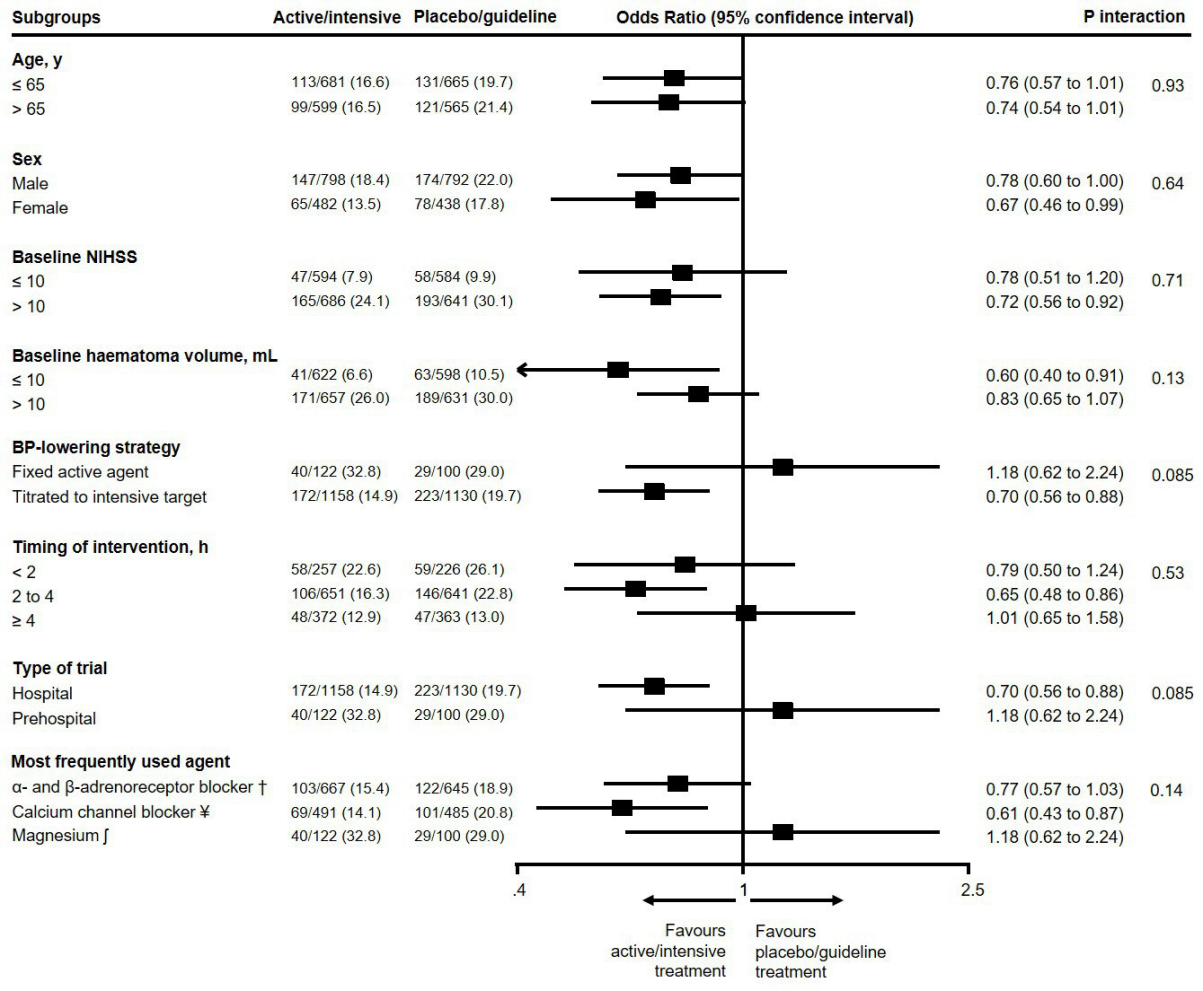
Effects of blood pressure (BP) lowering interventions in active/intensive versus placebo/guideline groups after acute intracerebral haemorrhage (ICH) on absolute haematoma growth ≥6 mL at 24 hours in subgroups. Model adjusted for age, sex, baseline haematoma volume and time from symptom onset to randomisation. †INTERACT1&2, ICH-ADAPT; ¥ATACH-II; ʃFAST-MAG.

There were 2510 patients from 5 trials with complete IPD for adjusted analysis of the secondary outcome of haematoma growth at 24 hours (table 2). The absolute difference in haematoma growth at 24 hours between active/intensive BP management and placebo/guideline BP management was −1.10 mL (−2.22 to 0.01) mL, p=0.05. Compared with placebo/guideline BP management, active/intensive BP-lowering interventions reduced the odds of haematoma growth, when assessed either by an absolute (>6 mL, adjusted OR 0.75, 95% CI 0.60 to 0.92; p=0.0077) or relative (≥33%, adjusted OR 0.82, 95% CI 0.68 to 0.99; p=0.034) increase between baseline and 24 hours. There was no heterogeneity in the treatment effect across prespecified subgroups (figure 5).

## Discussion

In this meta-analysis of patient-level data from RCTs of various BP-lowering interventions in adults with predominantly mild-to-moderate severity acute ICH, we have shown that a moderate degree of BP lowering does not improve functional outcome by 3–6 months. However, BP lowering can reduce the risk of haematoma growth—a factor strongly associated with poor outcome^30 31^—in a similar manner according to patient age, sex and baseline haematoma volume. We have also provided new information about the potential for heterogeneity in the effects of BP lowering according to the treatment strategy and agent.

There has been uncertainty about the most effective strategy for lowering BP after acute ICH.^13 16^ Our study included patients treated with a broad range of interventions by which to make comparisons: in prespecified subgroup analysis, we found significant heterogeneity in the treatment effect according to the strategy used to deliver BP lowering. Patients who received BP-lowering interventions titrated to an intensive target had larger reductions in their systolic BP and seemed to have better functional outcome compared with patients treated with a fixed active agent. These findings are supported by previous observational analysis of pooled data from two RCTs (n=3829) which suggested that careful, targeted and sustained reductions in systolic BP (to as low as 120–130 mm Hg in the first 24 hours) are safe, and associated with better functional outcome.^4^

Uncertainty also persists about the most effective agent for lowering BP after acute ICH.^15 16^ Our study included patients who were treated with various BP-lowering agents that were delivered via intravenous, oral, sublingual and transdermal routes. Our prespecified subgroup analysis showed that the effects of interventions varied according to the categorisation of each trial by the most frequently used agent in the treatment arm, with patients treated in trials of predominantly intravenous α-adrenoreceptor and β-adrenoreceptor blockers appearing to have better functional outcome compared with patients treated with other agents. This might be explained by the potential for intravenous α-adrenoreceptor and β-adrenoreceptor blockers to target peripheral arteriolar resistance and attenuate the sympathetic response that has been shown to determine high admission BP after acute ICH.^32^ Their use may also avoid the potential deleterious effects of vasodilation associated with ultraearly use of topical nitrates.^33^ However, more data are needed: the recently initiated, INTEnsive ambulance-delivered blood pressure Reduction in hyper-Acute stroke (INTERACT4) trial, aims to determine if prehospital administered BP lowering with the intravenous α-adrenoreceptor blocker urapidil, improves outcome in patients with a diagnosis of ICH (ClinicalTrials.gov: NCT03790800).

The large pooled dataset also allowed us to test the ‘time is brain’ hypothesis in relation to BP lowering after ICH. Another IPD meta-analysis has shown that time to diagnostic imaging, baseline haematoma volume and anticoagulant use at symptom onset were the key predictors of subsequent haematoma growth: the earlier the scan and larger the haematoma, the greater the risk of growth, peaking at 0.5–3 hours after symptom onset and a volume of 75 mL, respectively.^24^ More recently, post hoc analyses of the ATACH-II trial have suggested a benefit of BP lowering in patients who received the treatment within 2 hours of symptom onset.^34^ In our study, where median time to randomisation and baseline haematoma volume were 3.8 hours and 10.7 mL, respectively, greater reductions in haematoma growth seemed to occur in patients in whom treatment was initiated between 2 and 4 hours compared with other time periods from symptom onset, but without there being statistical heterogeneity across the subgroups. The absence of effect in patients treated earlier may be due to a substantial proportion of patients in the <2 hours subgroup being from prehospital studies of intravenous magnesium (which had no demonstrable effect on BP) and topical nitrates (which may be harmful very early after ICH due to their vasodilatory properties^33^). Further research is awaited to validate previous findings about ultraearly nitrate use (Multicentre Randomised trial of Acute Stroke treatment in the Ambulance with a nitroglycerin Patch, MR ASAP, ISRCTN99503308) and between 3 and 5 hours after the onset of symptoms (Efficacy of Nitric Oxide in Stroke-2, EudraCT 2020-001304-42), and establish the safety of other agents in the prehospital setting (INTERACT4, NCT03790800).

Key strengths of this study are the broad inclusion criteria and availability of IPD from most high-quality ICH and mixed stroke trials in the area. This increased the sample of patients with ICH to 6221, compared with 4360 in a previous study-level meta-analysis of ICH studies only.^14^ The unique dataset facilitated robust covariable-adjusted analyses which provide reliable evidence about the effect of BP-lowering on ICH growth. The analyses were also adequately powered to detect heterogeneity in treatment effect according to various aspects of the BP-lowering interventions, including strategy, timing and most frequently used agent. We acknowledge that a lack of IPD from various small (n<250) studies raises the possibility of data availability bias, but many of these had a high risk of bias and reported outcomes that were not suitable for meta-analysis and thus, are unlikely to have influenced our overall results and conclusions. Although we had a reasonably large sample (n=2510) for analyses of the effects of BP lowering on haematoma growth, the majority of patients contributing data came from studies that tested early intensive, titrated target-based BP lowering. Other notable limitations fail to resolve uncertainty over the mechanisms underpinning the main findings: first, our assessment of drug-class effects was based on the most frequently used agent in the active/intensive treatment group of each trial; the frequent use of rescue agents/multiagent regimens for resistant hypertension limits the reliability of any conclusions made about a preferred agent. Second, despite the benefits of a large sample size and greater statistical precision, the current analyses may have failed to detect complex multilevel interactions between setting, timing and strategy. Third, our analyses do not account for the speed and degree of BP lowering, where the potential risks (end-organ ischaemia) and benefits (reductions in haematoma growth) of treatment may be finely balanced. Finally, we were unable to adjust for other potential confounders of outcome after acute ICH including race/ethnicity, time to drug administration, withdrawal of active care^35^ and poor baseline kidney function^36^ as these variables were not acquired from several studies.

In summary, our findings reinforce complexities to the generation of evidence over treatments for acute ICH. We have shown that while moderate BP reduction, applied within several hours of the onset of ICH, is safe and reduces the likelihood of growth of small-to-medium sized haematomas, this does not clearly translate into improved odds of recovery. Subgroup analyses suggest that BP-lowering interventions may be more effective if they are titrated to an intensive target rather than applied as a fixed dose of an active agent, or involve drugs with preferential α-adrenoreceptor and β-adrenoreceptor blockade rather than involving calcium channel blockers, nitrates or angiotensin system blockers.

## Data Availability

Data are available in a public, open access repository. Data are available on reasonable request. Requests for sharing of de-identified IPD from individual trials used in these analyses should be directed to the corresponding author of the individual trial. The ATACH-II trial data, including de-identified participant data, are available indefinitely at the National Institute of Neurological Disorders and Stroke data archive (https://www.ninds.nih.gov/). To gain access, requesters will need to sign a data-access agreement.
