# Supplementary material for: Early lowering of blood pressure after acute intracerebral haemorrhage: a systematic review and meta-analysis of individual patient data
Source: J Neurol Neurosurg Psychiatry. 2021 Nov 3;93(1):6–13. doi: 10.1136/jnnp-2021-327195 (PMC8685661; doi:10.1136/jnnp-2021-327195)
Supplement: Supplementary data [file jnnp-2021-327195supp001.pdf]

SUPPLEMENTARY MATERIALS

Title: Early lowering of blood pressure after acute intracerebral haemorrhage: a systematic review and meta-analysis of individual patient data

The Blood Pressure in Acute Stroke (BASC) Investigators*

*Writing Committee: Tom J Moullaali Ph.D†,[1,2] Xia Wang Ph.D†,[1] Else Charlotte Sandset Ph.D†,[3,4] Lisa J Woodhouse MSc†,[5] Zhe Kang Law Ph.D†,[5,6,7] Hisatomi Arima Ph.D,[8] Kenneth S Butcher Ph.D,[9] John Chalmers Ph.D,[1] Candice Delcourt Ph.D,[1,10,11] Leon Edwards,[10,11] Salil Gupta M.D,[12] Wen Jiang Ph.D,[13,14] Sebastian Koch M.D,[15] John Potter FRCP,[16,17] Adnan I Qureshi M.D,[18] Thompson G Robinson M.D,[19] Rustam Al-Shahi Salman Ph.D,[2] Jeffrey L. Saver M.D,[20] Nikola Sprigg FRCP,[5,6] Joanna Wardlaw F.Med.Sci,[2] Craig S Anderson Ph.D‡,[1,9,10,21] Philip M Bath F.Med.Sci‡.[5,6]

[1]The George Institute for Global Health, Faculty of Medicine, University of New South Wales, NSW, Australia

[2]Centre for Clinical Brain Sciences, University of Edinburgh, University of Edinburgh, Edinburgh, UK

[3]Department of Neurology, Oslo University Hospital, Oslo, Norway

[4]Research and Development Department, The Norwegian Air Ambulance Foundation, Oslo, Norway

[5]Stroke Trials Unit, Division of Clinical Neuroscience, University of Nottingham, Nottingham, UK

[6]Stroke, Nottingham University Hospitals NHS Trust, Nottingham, UK

[7]National University of Malaysia, Kuala Lumpur, Malaysia

[8]Department of Preventive Medicine and Public Health, Fukuoka University, Fukuoka, Japan

[9]Prince of Wales Clinical School, University of New South Wales, Randwick, New South Wales, Australia

[10]Neurology Department, Royal Prince Alfred Hospital, Sydney, Australia

[11]Central Clinical School, the University of Sydney, Sydney, NSW, Australia

[12]Department of Neurology, Army Hospital Research and Referral, New Delhi, India

[13]Department of Neurology, Xijing Hospital, Fourth Military Medical University, Xi'an, China

[14]The Shaanxi Cerebrovascular Disease Clinical Research Center, Xi'an, China

[15]Department of Neurology, University of Miami Miller School of Medicine, Miami, FL, United States

[16]Stroke Research Group, Norfolk and Norwich University Hospital, UK

[17]Norwich Medical School, University of East Anglia, UK

[18]Zeenat Qureshi Stroke Institute and Department of Neurology, University of Missouri, Columbia, MO

Moullaali TJ, *et al. J Neurol Neurosurg Psychiatry* 2021; 93:6–13. doi: 10.1136/jnnp-2021-327195

[19]University of Leicester, Department of Cardiovascular Sciences and NIHR Leicester Biomedical Research Centre, Leicester, UK

[20]Department of Neurology and Comprehensive Stroke Center, UCLA, Los Angeles, USA

[21]The George Institute China at Peking University Health Science Center, Beijing, PR China

†Contributed equally

‡Contributed equally

Corresponding Author:

Professor Craig S Anderson

The George Institute for Global Health

PO Box M201, Missenden Road, NSW 2050, AUSTRALIA

T: +61-2-9993-4500; F: +61-2-9993-4502; E: canderson@georgeinstitute.org.au

SUPPLEMENTARY TABLE 1 Comparison of one- and two-stage approaches to individual patient data (IPD) meta-analysis

**One-stage approach**	**Two-stage approach**
IPD from all studies are analysed together as if they belonged to a single study, adjusted for covariables. Statistical clustering can be addressed with multi-level modelling, where the source trial is treated as a random effect.	IPD from each study are analysed separately, adjusted for covariables. Then, similarly to aggregate-data meta-analysis, study-level estimates of effect are pooled in a random-effects meta-analysis model.

Moullaali TJ, *et al. J Neurol Neurosurg Psychiatry* 2021; 93:6–13. doi: 10.1136/jnnp-2021-327195

SUPPLEMENTARY TABLE 2 Design characteristics of eligible studies that provided individual patient data

**Study (N=16)**	**Design**	**Phase**	**Sites (N)**	**ICH (N=6221)**	**SBP inclusion (mmHg)**	**Time window (hours)**	**Intervention**	**Length of treatment (days)**	**Primary outcome**	**Follow-up (days)**	**Outcome on mRS reported?**
**ICH only**											
ATACH-II[1]	PROBE	III	109	1000	>180	<3	Nicardipine iv vs placebo	1	mRS 4-6	90	Yes
ICH ADAPT[2]	PROBE	IIb	3	75	>150	<24	Target SBP <150 vs <180 mmHg	1	Perihaematomal blood flow at 2h	90	Yes
Gupta 2018[3]	PRO (not blind endpoint)	N/A	3	118	N/A	<72	Treat if MAP >=115 vs >=130	3	mRS 3-6	90	Yes
INTERACT1[4]	PROBE	IIc	44	404	150-220	<6	Target SBP <140 vs <180 mmHg	7	Proportional change in ICH volume at 24h	90	Yes
INTERACT2[5]	PROBE	III	170	2829	150-220	<6	Target SBP <140 vs <180 mmHg	1	mRS 3-6	90	Yes
Koch 2008[6]	PROBE	N/A	1	42	MAP >110	<8	Target MAP<110 vs 110-130	2	Neurological deterioration (NIHSS) within 48h	90	Yes
**Mixed stroke**											
CHHIPS[7]	DBPC	III	6	25	>160	<36	Labetalol 50mg po/im or lisinopril po/sl 5mg vs placebo	14	mRS 4-6	14 (90)	Yes

Study (N=16)	Design	Phase	Sites (N)	ICH (N=6221)	SBP inclusion (mmHg)	Time window (hours)	Intervention	Length of treatment (days)	Primary outcome	Follow-up (days)	Outcome on mRS reported?
CHASE[8]	PROBE	N/A	26	242	150-220	<72	10-15% reduction in SBP vs <180	7	mRS 3-6	90	Yes
ENOS (GTN)[9]	SBBE	III	168	629	140-220	<48	GTN 5mg td vs none	7	mRS (ordinal)	90	Yes
GTN-1[10]	DBPC	IIa	1	4	N/A	<120	GTN 5mg td vs placebo	12	mRS 3-6	90	Yes
GTN-2[11]	SBBE	IIb	1	5	100-230	<72	GTN 10mg vs 5mg td vs none	10	BP on day 1	90	Yes
SCAST[12]	DBPC	III	146	274	>140	<30	Candesartan po vs placebo	7	Vascular death, MI, or stroke; mRS (ordinal)	180	Yes
VENUS[13]	DBPC	III	?	35	130-220	<6	Nimodipine 120mg po vs placebo	10	mRS 4-6	90	Yes
**Pre-hospital**											
FAST-MAG[14]	DBPC	III	45	387	N/A	<2	Magnesium * iv vs placebo	1	mRS (ordinal)	90	Yes
RIGHT-1[15]	SBBE	IIa	1	6	>140	<4	GTN 5mg td vs none	7	SBP at 2h	90	Yes
RIGHT-2[16]	DBPC	III	54	145	>120	<4	GTN 5mg td vs sham	4	mRS (ordinal)	90	Yes

PROBE denotes prospective randomised open-label blinded-endpoint; DBPC, double-blind placebo-controlled; SBBE, single blind blinded endpoint, MAP, mean arterial pressure; SBP, systolic blood pressure; iv, intravenous; po, oral; sl, sublingual; td, transdermal; im: intramuscular; GTN, glyceryl trinitrate; ICH, intracerebral haemorrhage; mRS, modified Rankin Scale; NIHSS, National Institutes for Health Stroke Scale score

SUPPLEMENTARY TABLE 3 Design characteristics of eligible studies that did not provide individual patient data

**Study (N=34)**	**Design**	**Phase**	**Sites (N)**	**ICH (N=5273)**	**SBP inclusion (mmHg)**	**Time window (hours)**	**Intervention**	**Length of treatment (days)**	**Primary outcome**	**Follow-up (days)**	**Outcome on mRS reported?**
**ICH only**											
Chen 2010[17]	PROBE?	N/A	1	146	MAP >130	<6	MAP (150/90) 110 vs 130 (180/105) urapadil	1	Change in ICH volume at 24h	30	No
Dai 2005[18]	PROBE?	N/A	1	85	>180/100	<6	Nimodipine vs control	1	Nervous function defect	90	No
Duan 2011[19]	PROBE?	N/A	1	91	>180/100	<6	Target 130-150/90-100 vs 180/105	1	Proportional change in ICH volume at 24h	1	No
Gong 2013[20]	PROBE?	N/A	1	120	>160	<4	Target SBP <140 vs <180 using GTN	1	Change in ICH volume at 24h	14	No
Gong FT 2015[21]	PROBE?	N/A	1	120	>160	<4	Target SBP 130-140 vs 160-180 using GTN	1	Change in ICH volume at 24h	14	No
Guo 2016[22]	PROBE?	N/A	1	100	?	<3	Target SBP 130-140 vs 160-180	7	?	90	?
Huo 2012[23]	PROBE?	N/A	1	124	NA	<6	SBP 120-150 vs 150-180	1	Change in ICH volume at 48h	30	No
Jiang AC 2013[24]	PROBE?	N/A	1	106	>180 or MAP >140	<6	Target SBP <140 vs <180	1	Proportional change in ICH volume at 24h	14	No

Study (N=34)	Design	Phase	Sites (N)	ICH (N=5273)	SBP inclusion (mmHg)	Time window (hours)	Intervention	Length of treatment (days)	Primary outcome	Follow-up (days)	Outcome on mRS reported?
Jiang M 2013[25]	PROBE?	N/A	1	96	>180/100	<6	160-180/90-100	1	Change in ICH volume at 24h	21	No
Kan 2020[26]	PROBE?	N/A	?	1500	?	?	?	?	Several stated, without clear primary: ICH volume, adverse events, mRS	?	Yes
Lee JP 2011[27]	PROBE?	N/A	1	138	>180/110	<24	160 -180 /90 - 100 vs ~180/105	1	Change in ICH volume at 24h	1	No
Lee L 2011[28]	PROBE?	N/A	1	64	>170	<24	Target <140 vs 170-220	1	Change in ICH volume at 24h	28	No
Lee YB 2013[29]	PROBE?	N/A	1	78	>180/100	<6	Target 130-150/90-100 vs 180/105	1	Change in ICH volume at 72h	30	No
Liang 2013[30]	PROBE?	N/A	1	178	>170	<24	Target SBP <140 vs <170 mmHg	1	Change in ICH volume at 24h	28	No
Luo 2010[31]	PROBE?	N/A	1	122	N/A	<12	Target SBP <140 vs <180	1	Change in ICH and edema volume at 7, 28, and 90d	90	No
Ma 2013[32]	PROBE?	N/A	1	160	>180/110	<24	130-150/80-90 vs 180/105	1	Change in ICH volume at 24h	1	No
Song 2010[33]	PROBE?	N/A	1	256	>180/110	<24	intensive vs guideline	1	Change in ICH volume at 24h	21	No
Sun 2012[34]	PROBE?	N/A	1	120	>180/110	<24	Target SBP <140 vs <180	1	Change in ICH volume at 24h	1	No

**Study (N=34)**	**Design**	**Phase**	**Sites (N)**	**ICH (N=5273)**	**SBP inclusion (mmHg)**	**Time window (hours)**	**Intervention**	**Length of treatment (days)**	**Primary outcome**	**Follow-up (days)**	**Outcome on mRS reported?**
Szczechowski 1994[35]	?	N/A	?	17	?	<24	Nimodipine 1-2mg iv vs dextran 40,000	?	?	N/A	?
Tao 2014[36]	PROBE?	N/A	1	54	N/A	<3	Acupuncture vs control	1	Change in ICH volume at 24h	10	No
Wang 2012[37]	PROBE?	N/A	1	110	>180/110	<24	160-180/90-100	1	Change in ICH volume at 24h	21	No
Xian 2010[38]	PROBE?	N/A	1	106	>180/105	<72	140/90 vs 170/100	1	Change in ICH volume at 24h	90	No
Xu Bo 2010[39]	PROBE?	N/A	1	400	150-220	<6	Target SBP <140 vs <180 using urapadil	2	Change in ICH volume at 24h	3	No
Xu MY 2011[40]	PROBE?	N/A	1	41	150-220	<6	Target SBP <140 vs <180	1	Change in ICH volume at 24h	90	No
Zang 2019[41]	PROBE?	N/A	1	121	150-220	<5	Target 135-140 where SBP>150 vs <140 where SBP>180	3	ICH volume at 24h	90	Yes
Zhang HX 2011[42]	PROBE?	N/A	1	121	NA	<6	Target SBP <140 vs <180 using sodium nitroprusside	1	Change in ICH and edema volume at 24 and 72h, 7 and 14d	30	No
Zhang SJ 2011[43]	PROBE?	N/A	1	112	>180/110 or MAP >130	<24	Target 130-150/80-90 vs 180/105	1	Change in ICH volume at 24h	21	No

**Study (N=34)**	**Design**	**Phase**	**Sites (N)**	**ICH (N=5273)**	**SBP inclusion (mmHg)**	**Time window (hours)**	**Intervention**	**Length of treatment (days)**	**Primary outcome**	**Follow-up (days)**	**Outcome on mRS reported?**
Zhang HT 2016[44]	PROBE?			102		<48	Target 130-140 vs 170-180	1	Change in ICH volume at 48h	30	No
Zhao 2012[45]	PROBE?	N/A	1	76	>180/100	<6	Target 150/90 vs 165-180/95-100	1	Change in ICH volume at 24h	1	No
Zheng 2017[46]	PROBE	II	1	201	150-220	<24	Target 120-140 vs 140-180	7	rehaemorrhage at 7 days after surgery	90	No
**Mixed**											
IMAGES-pilot[47]	DBPC	IIb	1	6	N/A	<12	Magnesium 73 mmol iv vs placebo	1	Barthel <60	90	?
IMAGES[48]	DBPC	III	99	200	N/A	<12	Magnesium 81 mmol iv vs placebo	1	Barthel <60 or mRS >1	90	Yes
Uzuner (unpublished)	?	?	?	?	?	?	Nimodipine * iv vs *	?	?	?	?
**Pre-hospital**											
PIL-FAST[49]	DBPC	IIa	1	3	>160	<3	Lisinopril 5-10mg sl vs placebo	7	Feasibility data	7	No

PROBE denotes prospective randomised open-label blinded-endpoint; DBPC, double-blind placebo-controlled; SBBE, single blind blinded endpoint, MAP, mean arterial pressure; SBP, systolic blood pressure; iv, intravenous; po, oral; sl, sublingual; td, transdermal; im: intramuscular; GTN, glyceryl trinitrate; ICH, intracerebral haemorrhage; mRS, modified Rankin Scale; NIHSS, National Institutes for Health Stroke Scale score

SUPPLEMENTARY TABLE 4 Bias assessment

	Risk of bias domains						
	D1	D2	D3	D4	D5	D6	Overall
Anderson 2008 (INTERACT1)	+	+	-	+	+	-	+
Anderson 2013 (INTERACT2)	+	+	-	+	+	+	+
Ankolaker 2012 (RIGHT-1)	?	+	-	+	+	+	-
Bath 2001(GTN-1)	+	+	+	+	+	?	+
Bath 2015 (ENOS)	+	+	-	+	+	+	+
Bath 2019 (RIGHT-2)	+	+	+	+	+	+	+
Butcher 2013 (ICH-ADAPT)	+	+	-	+	+	+	+
Chen 2010	?	?	-	?	+	+	X
Dai 2005	?	?	-	?	+	?	X
Duan 2011	?	?	-	?	+	+	X
Gong 2013	+	?	-	?	+	+	X
Gong 2015	?	?	-	?	+	+	X
Guo 2016	+	?	-	+	+	?	-

D1: Randomisation method
D2: Allocation concealment
D3: Blinding of participants and personnel
D4: Blinding of outcome assessment
D5: Incomplete outcome data
D6: Selective reporting

X High
- Uncertain
+ Low
? No information

Gupta 2018	+	+	-	X	+	?	X
Kan 2020	?	?	?	?	?	?	?
Huo 2012	?	?	-	?	+	+	X
Horn 2001 (VENUS)	+	+	+	+	+	?	+
Jiang AC 2013	+	?	-	?	+	+	X
Jiang M 2013	?	?	-	?	+	+	X
Koch 2008	?	+	-	+	+	?	+
Lee JP 2011	?	?	-	?	+	+	X
Lee L 2011	?	?	-	?	+	+	X
Lee YB 2013	?	?	-	?	+	+	X
Lees 2004 (IMAGES)	+	+	+	+	+	+	+
Liang 2013	?	?	-	?	+	+	X
Luo 2010	+	?	?	?	+	+	X

D1: Randomisation method
D2: Allocation concealment
D3: Blinding of participants and personnel
D4: Blinding of outcome assessment
D5: Incomplete outcome data
D6: Selective reporting

X High
\- Uncertain
\+ Low
? No information

Ma 2013	?	?	-	?	+	+	X
Muir 1995 (IMAGES pilot)	+	+	+	+	+	?	+
Potter 2009 (CHHIPS)	+	+	+	+	+	+	+
Qureshi 2016 (ATACH-II)	+	+	-	+	+	+	+
Rashid 2003 (GTN-2)	+	+	-	+	+	?	+
Sandset 2011 (SCAST)	+	+	+	+	+	+	+
Saver 2015 (FAST-MAG)	+	+	+	+	+	+	+
Shaw 2013 (PIL-FAST)	+	+	+	+	X	+	-
Song 2010	?	?	-	?	+	+	X
Sun 2012	?	?	-	?	+	+	X
Szczechowski 1994	?	?	?	?	?	?	?
Tao 2014	+	?	-	?	+	+	X
Uzuner	?	?	?	?	?	?	?

D1: Randomisation method
D2: Allocation concealment
D3: Blinding of participants and personnel
D4: Blinding of outcome assessment
D5: Incomplete outcome data
D6: Selective reporting

X High
- Uncertain
+ Low
? No information

Moullaali TJ, *et al. J Neurol Neurosurg Psychiatry* 2021; 93:6–13. doi: 10.1136/jnnp-2021-327195

Wang 2012	?	?	-	?	+	+	X
Xian 2010	?	?	-	?	+	+	X
Xu 2010	+	?	-	?	+	+	X
Xu MY 2011	+	?	-	?	+	+	X
Yuan 2020 (CHASE)	+	+	-	+	+	+	+
Zhang HX 2013	+	?	-	?	+	+	X
Zhang SJ 2011	?	?	-	?	+	+	X
Zhang HT 2016	?	?	-	?	+	+	X
Zang 2019	?	?	-	?	+	?	X
Zhao 2012	?	?	-	?	+	+	X
Zheng 2017	?	?	-	?	+	+	X

D1: Randomisation method
D2: Allocation concealment
D3: Blinding of participants and personnel
D4: Blinding of outcome assessment
D5: Incomplete outcome data
D6: Selective reporting

X High
- Uncertain
+ Low
? No information

SUPPLEMENTARY TABLE 5 Post-hoc sensitivity analysis: effect of active/intensive vs. placebo/guideline blood pressure lowering interventions on functional outcome after acute intracerebral haemorrhage adjusted for baseline Glasgow Coma Scale score.

**Outcome:** unfavourable shift in modified Rankin Scale scores at 90 days, adjusted for pre-specified covariables* and:	**Adjusted OR* (95%CI)**	**p value**
**GCS score**	0.97 (0.88 to 1.06)	0.50
**NIHSS score**	0.97 (0.88 to 1.06)	0.44

CI denotes confidence interval; OR, odds ratio.

*Age, sex, time from onset to randomisation and trial (random effect).

References

1. Qureshi AI, Palesch YY, Barsan WG, et al. Intensive blood-pressure lowering in patients with acute cerebral haemorrhage. N. Engl. J. Med. 2016;375(11):1033–43.

2. Butcher K, Jeerakathil T, Emery D, et al. The intracerebral haemorrhage acutely decreasing arterial pressure trial: ICH ADAPT. Int. J. Stroke 2010;5(3):227–233.

3. Gupta S, Abbot AK, Srinath R, et al. Randomised trial to assess safety and clinical efficacy of intensive blood pressure reduction in acute spontaneous intracerebral haemorrhage. Med. J. Armed Forces India 2018;74(2):120–5.

4. Anderson CS, Huang Y, Wang JG, et al. Intensive blood pressure reduction in acute cerebral haemorrhage trial (INTERACT): a randomised pilot trial. Lancet Neurol. 2008;7(5):391–399.

5. Anderson C, Heeley E, Huang Y, et al. Rapid blood-pressure lowering in patients with acute intracerebral haemorrhage. N Engl J Med. 2013;368(25):2355–2365.

6. Koch S, Romano JG, Forteza AM, et al. Rapid blood pressure reduction in acute intracerebral haemorrhage: Feasibility and safety. Neurocrit. Care 2008;8(3):316–321.

7. Potter JF, Robinson TG, Ford GA, et al. Controlling hypertension and hypotension immediately post-stroke (CHHIPS): a randomised, placebo-controlled, double-blind pilot trial. Lancet Neurol. 2009;8(1):48–56.

8. Yuan F, Yang F, Zhao J, et al. Controlling Hypertension After Severe Cerebrovascular Event (CHASE): A randomised, multicenter, controlled study. Int. J. Stroke 2020;

9. Bath PMW, Woodhouse L, Scutt P, et al. Efficacy of nitric oxide, with or without continuing antihypertensive treatment, for management of high blood pressure in acute stroke (ENOS): a partial-factorial randomised controlled trial. Lancet 2015;385(9968):617–28.

10. Bath PM, Pathansali R, Iddenden R, Bath FJ. The effect of transdermal glyceryl trinitrate, a nitric oxide donor, on blood pressure and platelet function in acute stroke. Cerebrovasc. Dis. 2001;11(3):265–72.

11. Rashid P, Weaver C, Leonardi-Bee J, et al. The effects of transdermal glyceryl trinitrate, a nitric oxide donor, on blood pressure, cerebral and cardiac haemodynamics, and plasma nitric oxide levels in acute stroke. J. Stroke Cerebrovasc. Dis. 2003;12(3):143–51.

12. Sandset EC, Bath PMW, Boysen G, et al. The angiotensin-receptor blocker candesartan for treatment of acute stroke (SCAST): a randomised, placebo-controlled, double-blind trial. Lancet 2011;377(9767):741–750.

13. Horn J, De Haan RJ, Vermeulen M, Limburg M. Very Early Nimodipine Use in Stroke (VENUS): A randomised, double-blind, placebo-controlled trial. Stroke 2001;32(2):461–5.

14. Saver JL, Starkman S, Eckstein M, et al. Prehospital use of magnesium sulfate as neuroprotection in acute stroke. N. Engl. J. Med. 2015;372(6):528–536.

15. Ankolekar S, Fuller M, Cross I, et al. Feasibility of an ambulance-based stroke trial, and safety of glyceryl trinitrate in ultra-acute stroke: the rapid intervention with glyceryl trinitrate in hypertensive stroke trial (RIGHT). Stroke 2013;44(11):3120–3128.

16. Bath PM, Scutt P, Anderson CS, et al. Prehospital transdermal glyceryl trinitrate in patients with ultra-acute presumed stroke (RIGHT-2): an ambulance-based, randomised, sham-controlled, blinded, phase 3 trial. Lancet 2019;393(10175):1009–20.

17. Chen JC, Sun DJ, Ge ZL. Effects of intensively antihypertensive therapy by Urapidil on haematoma enlargement in patients with hypertensive intracerebral haemorrhage. J. Clin. Neurol. 2010;

18. Dai HY, Yang YS, Guo FQ, et al. Effects of nimodipine on nervous functions, ability of daily life and plasma neuron-specific enolase of patients with hypertensive cerebral haemorrhage. Chinese.Journal Clin. 2005;

19. Duan G. The effect of early blood pressure redcution on haematoma expansion in hypertensive intracerebral haemorrhage. Chinese J. Pract. Med. 2011;6(34):95–96.

20. Gong F, Li-ping Y, Xia L, et al. Effects of blood pressure control on haematoma expansion and neurological function in patients with ultra-early basal ganglia intracerebral haemorrhage. Clin. Med. China 2013;29(4):361–3.

21. Gong F, Yu L, Gong Y, et al. Blood pressure control in ultra-early basal ganglia intracerebral haemorrhage. In: European review for medical and pharmacological sciences. 2015 p. 412–415.

22. Guo Y, Wang H, Zhang C, et al. Effect of hyperacute intensive antihypertensive treatment on the prognosis of intracerebral haemorrhage in basal ganglia region. Chinese J. Cerebrovasc. Dis. 2016;13(10):516–521.

23. Huo Q, Wang J. The effect of early intensive blood pressure reduction on haematoma expansion and prognosis in 124 cases of hypertensive intracerebral haemorrhage. Med. J. Commun. 2012;26(5):457–8.

24. Jiang A, Zhang J, Yang G. Influence of the Early Antihypertensive Therapy on Haematoma Enlargement in Patients with Hypertension Intracerebral Haemorrhage. Med. Recapitul. 2013;19(23):4369–70.

25. Jiang M. Effect of Intensive Antihypertensive Treatment on Prognosis in Patients with Hypertensive Cerebral Haemorrhage. ChinJMAP 2013;30(7):785–8.

26. Kan S, Sun R, Chai S, et al. A clinical study on the association of clinical outcome and acute systolic blood pressure in cerebral haemorrhage patients. Int J Clin Pharmacol Ther 2020;58(3):146–154.

27. Lee J. Effect of early blood pressure reduction on haematoma expansion in patients with cerebral haemorrhage. China Pract. Med. 2011;6(9):114–5.

28. Lee L, Zhang J, Song Y. The efficacy of early blood pressure reduction in patients with hypertensive intracerebral haemorrhage. Mod. J. Integr. Tradit. Chinese West. Med. 2011;20(22):2787–8.

29. Lee Y, Deng F, Chen C. Intensified antihypertension and sedation of micro-pumped infusion of propofol and sodium nitroprusside in acute stage of cerebral haemorrhage. Anhui Med. Pharm. J. 2013;17(3):495–7.

30. Liang S. A study of effect of early blood pressure reduction on short-term outcome in hypertensive intracerebral haemorrhage. Mod. J. Integr. Tradit. Chinese West. Med. 2013;22(16):1775–8.

31. Luo Z, Ju D. Early Intensive Blood Pressure Lowering Treatment of Cerebral Haemorrhage. Pract. Clin. Med. 2010;11(9):10–12.

32. Ma S, Zhao J, Li J, Li S. Influence of early intensive antihypertensive treatment on haematoma enlargement in patients with hypertensive intracerebral haemorrhage. Chinese J. Pract. Nerv. Dis. 2013;16(7):19–20.

33. Song WG, Zhong JG, Xiao PR. Influence of early intensively antihypertensive therapy on haematoma enlargement in patients with hypertensive intracerebral haemorrhage. J. Clin. Neurol. 2010;

34. Sun Z. The effect of early blood pressure reduction on haematoma expansion in hypertensive

intracerebral haemorrhage. Chinese J. Pract. Nerv. Dis. 2012;15(3):55–56.

35. Szczechowski L, Wajgt A. Evaluation of treatment efficiency of acute haemispheric strokes using intravenous nimodipine infusions. Neurol. i Neurochir. Pol. 1994;

36. Tao WQ, Fang HY, Zou ZQ, et al. Impacts of acupuncture on blood pressure and haematoma in patients of cerebral haemorrhage at the early stage. Zhongguo Zhenjiu 2014;34(5):426–30.

37. Wang X. The effect of early blood pressure reduction on haematoma growth in hypertensive intracerebral haemorrhage. China Pract. Med. 2012;7(27):118–9.

38. Xian M, Lei J, Yuan L, Al E. Effect and Safety of Early Intensive Blood Pressure Reduction in Patients with Acute Cerebral Haemorrhage. J. Med. Forum 2010;31(1):20–22.

39. Xu B, Zhang M, Zhao J, He L. A comparative study of relationship between acute blood pressure control and haematoma expansion. Neural Inj. Funct. Reconstr. 2010;5(5):386–7.

40. Xu MY, Zhou JS. Effect of blood pressure lowering strategy on the enlargement of haematoma and clinical outcome in patients with acute intracerebral haemorrhage. Chinese J. Cerebrovasc. Dis. 2011;8(1):23–27.

41. Zang Y, Zhang C, Song Q, et al. Therapeutic effect of early intensive antihypertensive treatment on rebleeding and perihaematomal edema in acute intracerebral haemorrhage. J. Clin. Hypertens. 2019;21(9):1325–1331.

42. Zhang H, Gu F, Huang Y. The therapeutic effect of early intensive blood pressure control on the perihaematomal edema in acute intracerebral haemorrhage. Chinese J. difficult Complicat. cases 2011;10(12):894–6.

43. Zhang S, Feng G, Feng X. The effect of early intensive blood pressure reduction in hypertensive intracerebral haemorrhage. Chinese J. Coal Ind. Med. 2011;14(7):1002–3.

44. Zhang HT, Yu M, Ren YF, et al. Effect of different blood pressure control targets within 48 h after hypertensive cerebral haemorrhage on haematoma enlargement and prognosis. J. South. Med. Univ. 2016;36(12):1616–1620.

45. Zhao Y. An impact analysis of blood pressure reduction on early haematoma exoansion in intracerebral haemorrhage. Chinese Community Dr. 2012;14(323):63.

46. Zheng J, Lin S, Li H, et al. Perioperative antihypertensive treatment in patients of spontaneous intracerebral haemorrhage (PATICH): A clinical trial protocol. Contemp. Clin. Trials 2014;39(1):9–13.

47. Muir KW, Lees KR. A randomised, double-blind, placebo-controlled pilot trial of intravenous magnesium sulfate in acute stroke. Stroke 1995;26(7):1183–8.

48. Muir KW, Lees KR, Ford I, Davis S. Magnesium for acute stroke (Intravenous Magnesium Efficacy in Stroke trial): randomised controlled trial. Lancet 2004;363(9407):439–45.

49. Shaw L, Price C, McLure S, et al. Paramedic Initiated Lisinopril For Acute Stroke Treatment (PIL-FAST): Study protocol for a pilot randomised controlled trial. Trials 2011;12:152.
